# 138. Description of Repeat Carbapenem-resistant Enterobacterales (CRE) Submitted to a State Laboratory from the Same Individuals, Kentucky - December 2018 - March 2022

**DOI:** 10.1093/ofid/ofac492.216

**Published:** 2022-12-15

**Authors:** Kevin B Spicer, Marwa Elathamna, Rachel Zinner

**Affiliations:** Centers for Disease Control & Prevention, Shelbyville, Kentucky; Kentucky Department for Public Health, Division of Laboratory Services, Frankfort, Kentucky; Kentucky Department for Public Health, Division of Laboratory Services, Frankfort, Kentucky

## Abstract

**Background:**

CRE and carbapenemase-producing CRE (CP-CRE) can be persistently detected during screening in long-term care facilities. Re-identification of these organisms during normal care and movement through healthcare settings is less well described.

**Objective:** To describe repeated submission of CRE to a state laboratory, including persistence of detection and progression from colonization to infection.

**Methods:**

Submission of CRE isolates was requested from labs in late 2017 and became mandated by legislative regulation in December 2020. Isolates were tested at the Kentucky Division of Laboratory Services (DLS) for CP using the modified carbapenem inactivation method (mCIM), with follow-up testing for specific carbapenemases using the Cepheid Xpert Carba-R assay. Results were maintained in the DLS lab information system. Descriptive statistics were derived for testing results from repeat submissions.

**Results:**

From December 2018 through March 2022, 1,822 isolates were submitted to DLS from 1,498 distinct individuals; 324 repeat submissions were obtained from 214 individuals, with 141 individuals tested > 14 days after the initial result (Figure). Several individuals had > 2 submissions and some cultures had CRE both with and without CP identified. The same CP-CRE (species and mechanism) was identified from 42 individuals (29.8%) when tested > 14 days after initial identification (median 71 days, IQR 40-151, max 857). The same CRE (species) without CP was identified from 66 individuals (44.1%; median 111.5 days, IQR 55-237, max 676). Urine was the most common specimen source among resubmissions (67% of resubmissions). Of 47 individuals with a discrepant result (i.e., different species or mechanism) after > 14 days, only four had the same species identified initially as CP-CRE and then subsequently as non-CP-CRE. Ten individuals (7%) had screening and then clinical specimens with the same CP or species.

**Figure:**
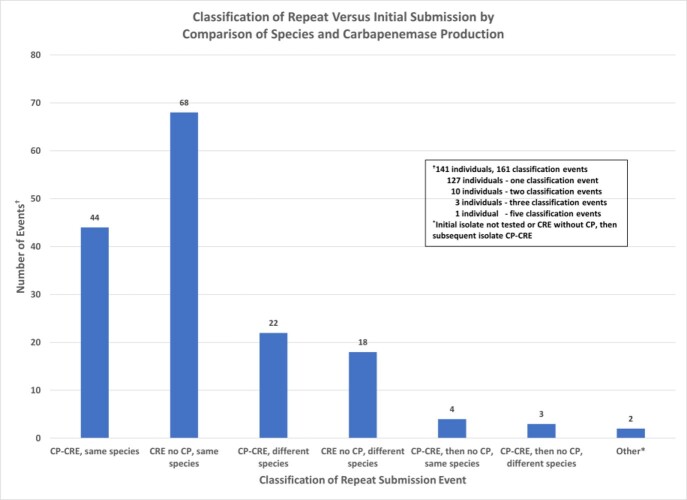

**Conclusion:**

Persistence can occur with CP-CRE and with CRE without CP and may continue for >500 days. The in vivo loss of CP by CRE appears to occur infrequently. Clinicians and infection preventionists should remain vigilant for persistent CRE colonization during clinical and infection control decision making.

**Disclosures:**

**All Authors**: No reported disclosures.

